# NLRP1 inflammasome involves in learning and memory impairments and neuronal damages during aging process in mice

**DOI:** 10.1186/s12993-021-00185-x

**Published:** 2021-12-17

**Authors:** Dan Sun, Guofang Gao, Bihua Zhong, Han Zhang, Shixin Ding, Zhenghao Sun, Yaodong Zhang, Weizu Li

**Affiliations:** 1 Department of Pharmacy, The First People’s Hospital of Xiaoshan District, 199 Shixin South Road, Hangzhou, 311200 Zhejiang China; 2 Department of Pharmacology, Basic Medicine College, Anhui Medical University, No. 81 Meishan Road, Hefei, 230032 Anhui China; 3 Key Laboratory of Anti-Inflammatory and Immunopharmacology, Ministry of Education, Anhui Medical University, No. 81 Meishan Road, Hefei, 230032 Anhui China

**Keywords:** Brain aging, Learning and memory impairments, NLRP1 inflammasome, ROS, NADPH oxidase 2

## Abstract

**Background:**

Brain aging is an important risk factor in many human diseases, such as Alzheimer’s disease (AD). The production of excess reactive oxygen species (ROS) mediated by nicotinamide adenine dinucleotide phosphate oxidase 2 (NOX2) and the maturation of inflammatory cytokines caused by activation of the NOD-like receptor protein 1 (NLRP1) inflammasome play central roles in promoting brain aging. However, it is still unclear when and how the neuroinflammation appears in the brain during aging process.

**Methods:**

In this study, we observed the alterations of learning and memory impairments, neuronal damage, NLRP1 inflammasome activation, ROS production and NOX2 expression in the young 6-month-old (6 M) mice, presenile 16 M mice, and older 20 M and 24 M mice.

**Results:**

The results indicated that, compared to 6 M mice, the locomotor activity, learning and memory abilities were slightly decreased in 16 M mice, and were significantly decreased in 20 M and 24 M mice, especially in the 24 M mice. The pathological results also showed that there were no significant neuronal damages in 6 M and 16 M mice, while there were obvious neuronal damages in 20 M and 24 M mice, especially in the 24 M group. Consistent with the behavioral and histological changes in the older mice, the activity of β-galactosidase (β-gal), the levels of ROS and IL-1β, and the expressions of NLRP1, ASC, caspase-1, NOX2, p47phox and p22phox were significantly increased in the cortex and hippocampus in the older 20 M and 24 M mice.

**Conclusion:**

Our study suggested that NLRP1 inflammasome activation may be closely involved in aging-related neuronal damage and may be an important target for preventing brain aging.

**Supplementary Information:**

The online version contains supplementary material available at 10.1186/s12993-021-00185-x.

## Introduction

Brain aging has been reported to be an important risk factor in many human diseases, such as Alzheimer’s disease (AD) and Parkinson’s disease (PD) [1]. At present, the mechanism of brain aging is still not completely understood. Growing evidence has indicated that neuroinflammation plays an important role in the aging process [2]. Even the regulation of neuroinflammation by the peripheral immune system is involved in the development of aging and AD [3]. According to a large amount of epidemiological, clinical, and laboratory data, the relationship between inflammation and aging-related diseases is inseparable [4]. It has been reported that IFN-γ and other proinflammatory cytokines interact with processing and production of Aβ peptide, suggesting that inflammation may be a "prodrome" to AD [4]. However, it is still unclear when and how the neuroinflammation takes place during aging process.

Inflammasomes are multiprotein complexes in the cytoplasm that are responsible for the formation of proinflammatory molecules. Inflammasomes have a central role in the inflammatory response and can be activated by diverse stimuli, leading to the maturation of proinflammatory cytokines [5]. The nucleotide-binding oligomerization domain NOD-like receptor protein 1 (NLRP1) is the first family of sensor proteins discovered to form inflammasomes. Recent studies demonstrated that the NLRP1 inflammasome is closely associated with neurological diseases such as AD [6]. NLRP1 immunopositive neurons were increased 25–30-fold in brains of AD patients compared to brains of normal elderly [7]. Our previous study showed that the NLRP1 inflammasome was significantly increased in primary hippocampal neurons with prolongation of culture time [8]. Down-regulation of the NLRP1 inflammasome improves cognitive deficits in different animal models [9]. NLRP1 inflammasome activation in hippocampal neurons significantly exacerbates age-related cognitive impairment [10]. However, it remains unclear when and how NLRP1 inflammasome is involved in aging-associated neuronal damage during aging process.

Reactive oxygen species (ROS) accumulation reportedly plays a crucial role in the induction of inflammatory cascades [11]. Excessive release of ROS further promotes neuronal damage and subsequent inflammation resulting in a feed-forward loop of neurodegeneration [12]. NADPH oxidase (NOX), a multi-protein enzyme, is currently the only enzyme family known to produce ROS as its sole function. It has been reported that NOX-mediated ROS production involves in NLRP3 activation in metabolic and cognitive diseases, such as type 2 diabetes mellitus, obesity, and AD [13]. Excessive NOX-derived ROS also contributes to neuronal loss via oxidative stress damage or disruption of redox signaling circuits [14]. The NOX family comprises membrane components, including p22phox and gp91phox homologues of NOX1–5, and several other cytosolic proteins, including p47phox, p40phox, and p67phox [15]. NOX2 is widely expressed throughout the brain in both microglia and neurons [16]. The NOX2 has been studied mainly in microglia, where it plays a role in inflammation and may also contribute to neuronal death in pathological conditions [16], but it is still not completely understood whether NOX2-derived ROS production involves in NLRP1 inflammasome activation during aging process.

In the present study, we hypothesized that NLRP1 inflammasome involves in aging-related neuronal damages and NOX2 plays an important role in NLRP1 inflammasome activation during aging process. To confirm our hypothesis, we investigated the learning and memory function, the changes of NLRP1 inflammasome activation, and the ROS production and NOX2 expression in the cortex and hippocampus in 6-, 16-, 20-, and 24-month-old (M) mice. The study has the potential to confirm when the neuroinflammation appears in the brain and whether NOX2-NLRP1 inflammasome is involved in aging-related neuronal damage during aging.

## Materials and methods

### Animals and treatment

A total of 48 male 6-month-old ICR mice weighing (33–40 g) were obtained from the Center of Laboratory Animals of Anhui Medical University (Hefei, China) at the same time. The animals were randomly divided into 4 groups (n = 12): 6 M, 16 M, 20 M, and 24 M. The control was 6 M group. All mice were housed (6 mice in each cage) in a pathogen-free, temperature-controlled room with a 12-h light/12-h dark cycle and unrestricted access to food and water. After a week of acclimatization, the 6 M group were sacrificed after behavioral tests for subsequent experiments. Animals in other groups were bred separately to 16 months, 20 months and 24 months old and used for experiments. The entire experiment lasted for 18 months. Three mice died at the age of 23–24 months. All procedures were performed based on the guidelines approved by the Animal Ethics and Care Committee of Anhui Medical University (LLSC20160183). The experimental procedure was showed in Additional file 1: Fig. S1.

### Open field test (OFT)

The OFT was performed to study changes of motor and exploratory behavior when the mice reached their specified age. The OFT equipment (Shanghai Biotechnology Co., Ltd.) comprised a computer-tracked cage (60 × 60 × 50 cm) divided into nine squares (one central and eight peripheral) by two perpendicular transverse lines and vertical lines as previously described [17]. Twelve mice in the 6 M, 16 M, and 20 M group and 9 mice in the 24 M group were allowed 24 h to adjust the environment. For the OFT, each mouse was placed in the cage for 2 min to adapt to the environment. Then, the motor and exploration paths were recorded for 3 min by ANY-maze Behavioral Tracking Software (Stoelting Co., Wood Dale, IL, USA). The total moving distance (m), the mean speed (m/s), the number of lines crossing, and the number of standing were calculated by the software to evaluate the motor and exploration behavior [18].

### Morris water maze (MWM)

The next day after the OFT, the mice were subjected to the MWM (in the same room with OFT) to detect learning and memory functions. The MWM test included four consecutive daily training trials and a spatial probe trial on the fifth day [19]. The test pool (120 cm in diameter and 60 cm high) filled with water (depth 30 cm, temperature 24 ± 2 °C) was divided into four quadrants. There was a hidden escape platform (9 cm in diameter) that was submerged 1 cm below the surface of the water in the third quadrant. For training trials, the mice with head facing towards the wall were individually placed into the tank from the four quadrants each day, each mouse was allowed to climbed onto the platform within 60 s. If the mice failed to find the hidden platform within 60 s, they were gently guided to the platform by the experimenter. Animals were allowed to remain there for 10 s to familiarize the location of the platform every time after climbing the platform. Each mouse underwent four trials per day, and the delay between trial is at least 20 min. The mean escape latency (MEL, s) of each day was recorded to evaluate learning ability. On the fifth day, the platform was removed from the pool, and each mouse performed a spatial probe test for 60 s. The latency of first entry to the platform (LFP, s), the swimming time in the quadrant of the platform (STP, s), and the number of crossing the platform (NCP) were recorded to indicate the memory results. At the end of each test, remove the mouse from the pool, wipe dry and return it to the home cage. During the test, keep the position of the spatial reference objects such as lights and objects in the laboratory unchanged, and eliminate the influence of interference factors on the experimental results.

### Histological examination

After behavior tests, the mice (n = 4) were killed by cervical dislocation, the brains were removed and fixed in 4% paraformaldehyde. The brain tissues were embedded in paraffin and sliced into 5 μm sections using a microtome (Leica, Nussloch, Germany). The sections were stained with hematoxylin and eosin (H&E) or Nissl staining (Beyotime Institute of Biotechnology, China) to examine the neuronal morphology in the cortex and hippocampal CA1 and CA3 regions using a microscope (Olympus IX71; Olympus, Tokyo, Japan). Nissl bodies appear blue-purple, and Nissl staining is often used to show the Nissl bodies in neurons. The density of the Nissl bodies was analyzed double-blindly from three random fields (400 ×) in cortex, hippocampal CA1 and CA3 areas by using the Image-Pro Plus 6.0 automatic analysis system to assess the amount of Nissl bodies [20].

### Determination of ROS production

The ROS level was evaluated by using dihydroethidium (DHE) fluorescence staining as previously described [21]. Briefly, DHE (Beyotime Biotechnology, Shanghai, China) was injected via the tail vein (100 μM, 0.1 ml/10 g body weight) for 30 min. Then the mice (n = 3) were sacrificed by cervical dislocation, and the brains were carefully removed and embedded in OCT (Sakura Finetek, Torrance, CA, USA). The brains were sectioned into 10 μm slices at − 20 °C with a freezing microtome (Leica CM3050, Nussloch, Germany). The sections were mounted onto slides and washed three times with PBS. Then, the sections were incubated with Hoechst 33258 (Sigma, 5 mg/L) for 5 min and washed three times with phosphate-buffered saline (PBS). The sections were examined using a fluorescence microscope (Olympus IX71) and photographed for DHE (excitation: 480 nm, emission: 590 nm) and Hoechst 33258 (excitation: 360 nm, emission: 450 nm). The mean fluorescence density of the DHE staining was quantified double-blindly from three random fields (400 ×) in cortex, hippocampal CA1 and CA3 areas by using Image-Pro Plus 6.0 automatic analysis system to assess the ROS production.

### Determination of senescence-associated β-galactosidase (β-gal) activity

OCT-embedded brains (n = 3) were cut into 10 µm sections. The sections were stained with a β-gal kit (Beyotime Biotechnology, Shanghai, China). All of the operations were performed strictly in accordance with the instructions. β-gal staining will generate blue products under the catalysis of senescence-associated β-gal with X-gal as the substrate. Thus, the density of blue cells can be used to examine the β-gal activity. The results were observed under a light microscope (Olympus IX71). The density was analyzed double-blindly from three random fields (400 ×) in cortex, hippocampal CA1 and CA3 areas by using Image-Pro Plus 6.0 automatic analysis system.

### Immunohistochemistry

For immunohistochemical staining, paraffin-embedded brain sections (n = 4) were deparaffinized and then were incubated with 3% H_2_O_2_ for 15 min at room temperature to remove the endogenous peroxidase activity. Subsequently, the sections were heated in a microwave oven with a sodium citrate antigen retrieval solution for 15 min and were blocked with non-immune goat serum for 30 min at 37 °C. Thereafter, the sections were washed with PBS and then incubated with a mouse anti-MAP2 antibody (1:200; ab11268; Abcam, Cambridge, UK) overnight at 4 °C. The next day, the sections were washed with PBS three times and then incubated with a general secondary antibody (ZSGB-BIO; Beijing, China) at room temperature for 1 h. The sections were performed with a DAB kit (ZSGB-BIO; Beijing, China). Finally, the tissues were counterstained with hematoxylin and were viewed under a microscope (Olympus IX72). The density was analyzed double-blindly from three random fields (400 ×) in cortex, hippocampal CA1 and CA3 areas by using Image-Pro Plus 6.0 automatic analysis system to indicate the changes of MAP2 expression during the aging process.

### Immunoblot analysis

Immunoblotting was performed according to previous description [22]. The hippocampal tissues (n = 3) were homogenized in radioimmunoprecipitation assay (RIPA) buffer (Beyotime Biotechnology, Shanghai, China) with protease and phosphatase inhibitors to extract total protein. The BCA Protein Assay Kit (Beyotime Biotechnology, Shanghai, China) was used to determine protein concentration. An equal amount (30 μg) of protein from each sample was separated by using sodium dodecyl sulfate–polyacrylamide gel (10%) electrophoresis (SDS-PAGE). The separated protein was transferred to a polyvinylidene fluoride (PVDF) membrane (Millipore, Bedford, MA, USA). The membranes were immersed in blocking buffer (5% fat-free milk in Tris-buffered saline with 20% Tween [TBST]) for 1 h, then incubated with primary antibodies against NLRP1 (1:1000, Abcam, ab3683), apoptosis-associated speck-like protein containing a caspase recruitment domain (ASC; 1:500, Santa Cruz Technology, SC-514414), caspase-1 (1:1000, Abcam, ab1872), interleukin (IL)-1β (1:1000, Abcam, ab9722), NOX2 (1:1000, Abcam, ab31092), p22phox (1:500, Bioworld Technology, BS60290), p47phox (1:1000, Bioworld Technology, BS4852), or β-actin (1:1000; ZSGB-BIO, TA-09) overnight at 4 °C. The next day, the membranes were incubated with horseradish peroxidase–conjugated secondary antibody (ZSGB-BIO, ZF-2301, 1:10,000) for 1 h at room temperature (24 °C). After washing three times with TBST, the proteins were visualized by an enhanced chemiluminescent reagent (Amersham Biosciences, UK). Images of the blots were obtained using a Chemi Q4800 mini-imaging system (Shanghai Bioshine Technology, Shanghai, China). The density of the protein band was measured using Image J 1.44 software; the density was then normalized to β-actin band. The relative density compared to the control group was calculated to indicate the expression of the target protein.

### Statistical analysis

All data are presented as mean ± standard deviation (SD). Statistical analyses were performed using SPSS 17.0 statistical software. The data were analyzed with one-way global analysis of variance (ANOVA). If it shows significant effect, then Bonferroni’s post hoc test is used to compare differences between groups. Statistical significance is defined as *P* < 0.05.

## Results

### Effects of aging on motor and exploratory behavior in mice

**Fig. 1:**
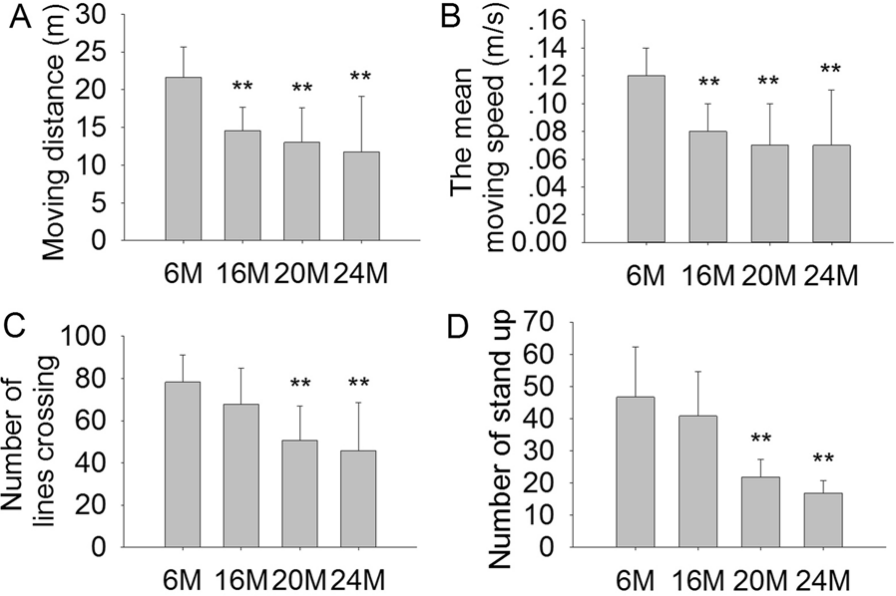
Effects of aging on motor activity and exploratory behavior in 6 M, 16 M, 20 M and 24 M mice (open field test). **A** The total moving distance (m). **B** The mean moving speed (m/s). **C** The number of lines crossing. **D** The number of standing. Results are expressed as mean ± SD. 6 M, 16 M, 20 M, n = 12; 24 M, n = 9. **P < 0.01 vs 6 M control

In the study, the OFT was used to observe the effects of aging on motor activity and exploratory behavior in mice. The results showed that the mean moving distance (m) (Fig. 1A; F(3,41) = 7.698, *P* < 0.01), the mean moving speed (m/s) (Fig. 1B; F(3,41) = 7.685, *P* < 0.01), the number of line crossing (Fig. 1C; F(3,41) = 7.035, *P* < 0.01), and standing up (Fig. 1D, F(3,41) = 15.72, *P* < 0.01) had significant effects, and they were significantly decreased in 20 M and 24 M mice compared to 6 M mice. In addition, the moving distance and the mean moving speed were significantly decreased, while the number of lines crossing and number of standing were not significantly decreased in 16 M compared to 6 M mice. These data demonstrated an aging phenomenon: 16 M mice showed a mild decrease in motor ability, while the effects were much more pronounced in 20 M and 24 M mice.

### Effects of aging on learning and memory abilities in mice

**Fig. 2:**
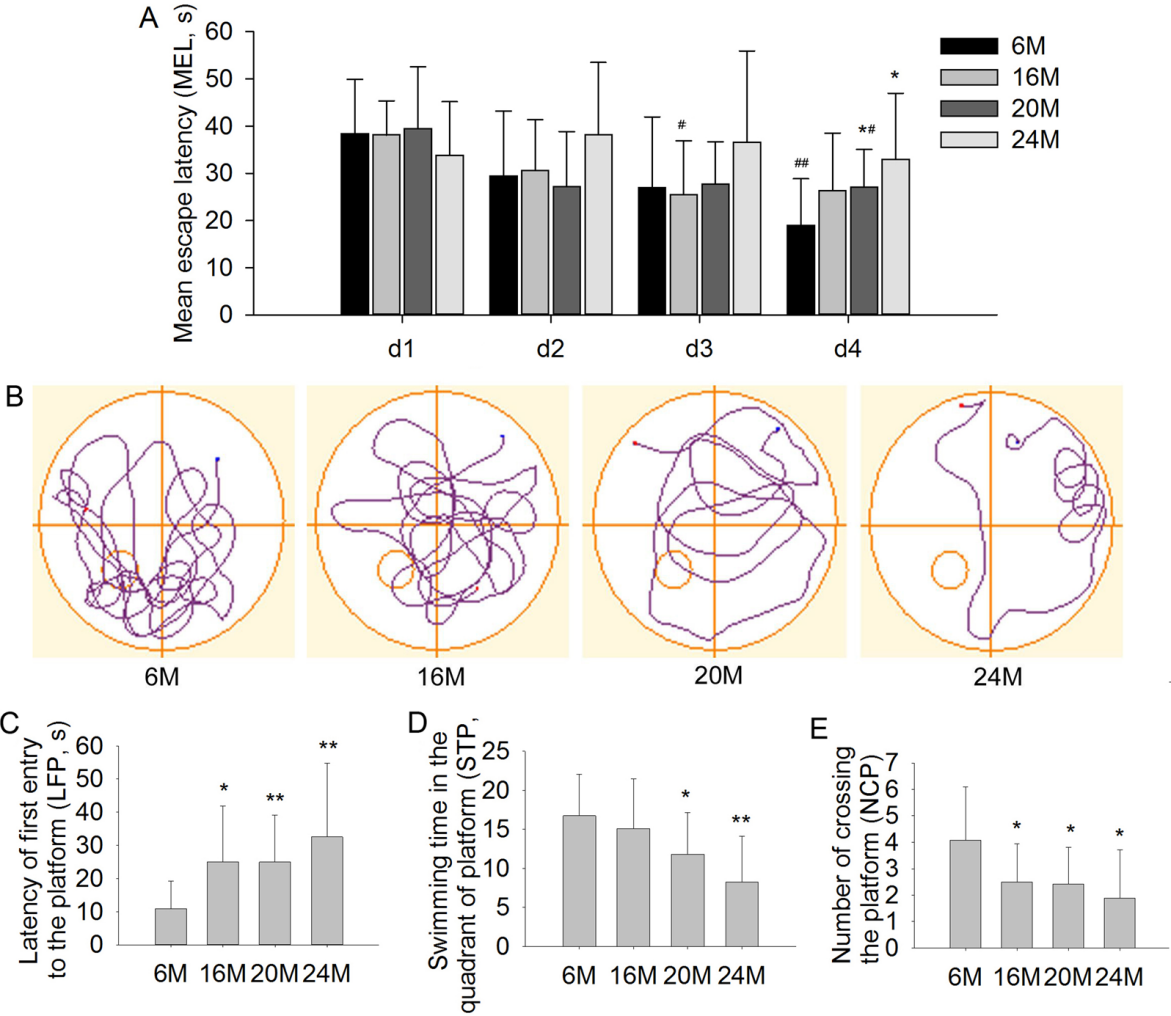
Effect of aging on learning and memory impairments in 6 M, 16 M, 20 M and 24 M mice (Morris water maze). **A** The mean escape latency (s) in the orientation navigation experiment. **B** Representative path of probe trial experiments on day 5. **C** The latency of first entry to the platform (s). **D** The swimming time in the quadrant of platform (s). **E** The number of crossing the platform. Results are expressed as mean ± SD. 6 M, 16 M, 20 M, n = 12; 24 M, n = 9. *P < 0.05, **P < 0.01 vs 6 M group; ^#^*P* < 0.05, ^##^*P* < 0.01 vs d1 in the orientation navigation experiment

The MWM was used to investigate changes in learning and memory abilities during the aging process in mice. In the orientation navigation experiment, compared with the first day (d1), the escape latency in 6 M, 16 M and 20 M group had a decreasing trend and significantly decreased on d4 in 6 M group (Fig. 2A; F(3,44) = 4.746, *P* < 0.01), on d3 in 16 M group (Fig. 2A; F(3,44) = 4.556, *P* < 0.05), and on d4 in 20 M group (Fig. 2A; F(3,44) = 3.885, *P* < 0.05). However, the escape latency in 24 M group had no decreasing trend from d1 to d4. Additionally, the escape latency had significant effects on d4 (Fig. 2A; F(3,41) = 3.57, *P* < 0.05). And compared with 6 M group, the escape latency was significantly prolonged in 20 M and 24 M mice on d4 (Fig. 2A; *P* < 0.05), and had no significant effects on d1-d3. In the space exploration experiment on d5, compared with 6 M mice, the latency of first entry to the platform (LFP, s) was significantly increased in 16 M, 20 M, and 24 M mice (Fig. 2B, C; F(3,41) = 3.617, *P* < 0.05 or *P* < 0.01). The swimming time in the quadrant with platform (STP, s) (Fig. 2D; F(3,41) = 4.416, *P* < 0.05 or *P* < 0.01) and the number of crossing the platform (NCP) (Fig. 2E; F(3,41) = 3.55, *P* < 0.05) were significantly decreased in 20 M and 24 M compared with 6 M mice. These results suggested that the learning and memory ability was mild decreased in 16 M mice, but was significantly impaired in 20 M and 24 M mice, especially in the 24 M group.

### Effects of aging on neuronal degeneration in the cortex and hippocampus in mice

H&E and Nissl staining were performed to examine neuropathological changes in the cortex and hippocampus during aging. Based on H&E staining, there were a few neuronal abnormalities in the cortex and hippocampal CA1 and CA3 regions in 6 M mice. Compared with 6 M mice, there were no obvious increase of pathological damages in 16 M mice. However, there were obvious pathological damages in the cortex and hippocampal CA1 and CA3 regions in 20 M and 24 M mice, especially in the 24 M group. More neurons exhibited nuclear pyknosis and hyperchromatic nuclei in cortex and CA3 regions in 24 M mice, and eosinophilic degeneration in CA3 region in 24 M mice (Additional file 2: Fig. S2).

**Fig. 3:**
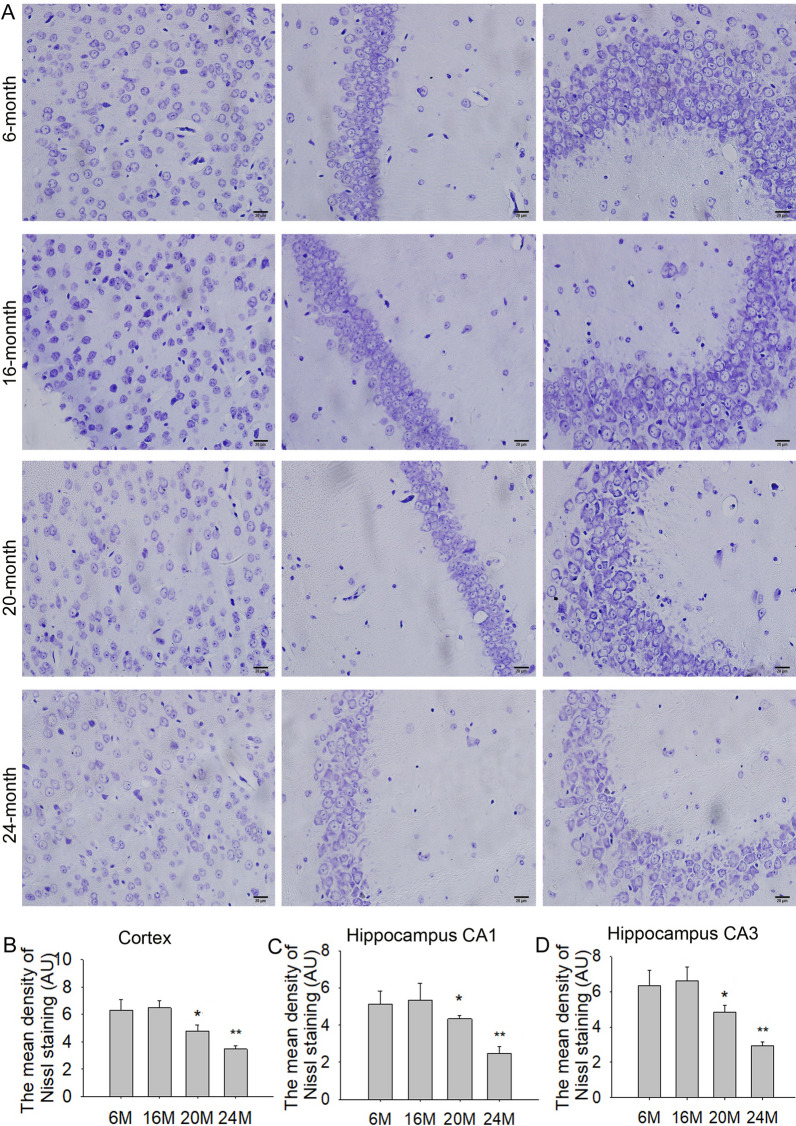
Effects of aging on changes of Nissl bodies in the cortex and hippocampus in mice (Nissl staining, 400 × , scale bar = 20 μm). **A** The results of Nissl staining in the cortex, hippocampus CA1 and CA3 in 6 M, 16 M, 20 M and 24 M mice. **B** The mean density of Nissl bodies in the cortex. **C** The mean density of Nissl bodies in hippocampus CA1. **D** The mean density of Nissl bodies in hippocampus CA3. Results are expressed as mean ± SD, n = 4. *P < 0.05, **P < 0.01 vs the 6 M control. AU presents an arbitrary unit

Nissl staining is a well-known method that specifically stains Nissl bodies and is often used to identify neuronal damage [23]. Nissl staining showed abundant Nissl bodies in the cortex and hippocampal CA1 and CA3 regions in 6 M and 16 M mice. When compared with 6 M mice, the number of Nissl bodies was significantly reduced in cortex (Fig. 3A, B; F(2,9) = 243.9, *P* < 0.01) and hippocampal CA1 (Fig. 3C; F(2,9) = 99.79, *P* < 0.05 or *P* < 0.01) and CA3 (Fig. 3D; F(2,9) = 71.02, *P* < 0.05 or *P* < 0.01) regions in 20 M and 24 M mice, especially the 24 M group. These results suggested that there were no significant neuronal damages before 16 M, but obvious neuronal damages were observed at the age of 20 M and 24 M in mice.

### Effects of aging on senescence-associated β-gal expression in the cortex and hippocampus in mice

**Fig. 4:**
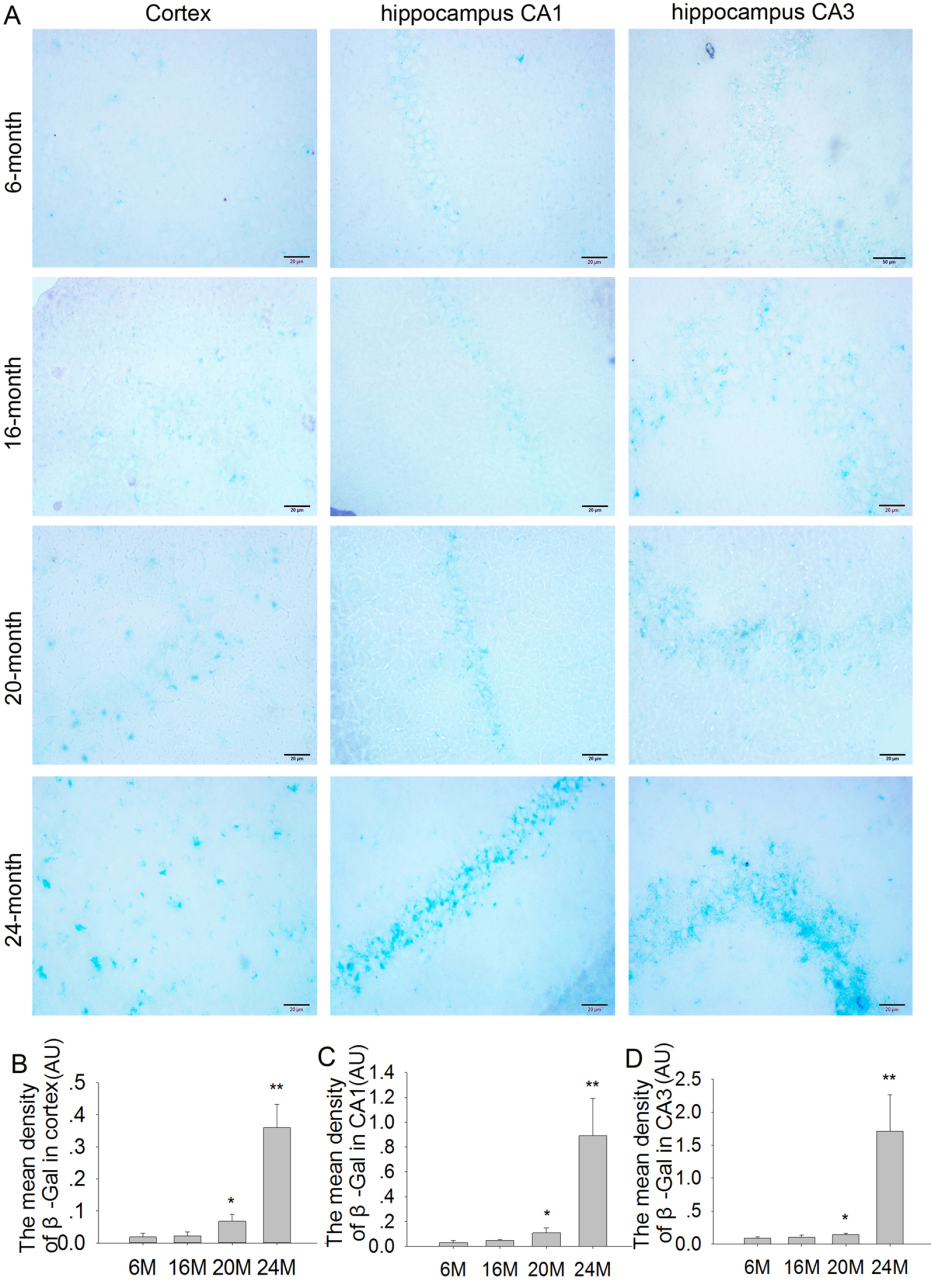
Effects of aging on β-gal activity in the cortex and hippocampus in mice. **A** The β-gal staining in the cortex, hippocampus CA1 and CA3 in the 6 M, 16 M, 20 M and 24 M mice (400 × , scale bar = 20 μm). **B** The mean density of β-gal in the cortex. **C** The mean density of β-gal in hippocampus CA1. **D** The mean density of β-gal in hippocampus CA3. Results are expressed as mean ± SD, n = 3. *P < 0.05, **P < 0.01 vs the 6 M control

The β-gal is an important biomarker for the senescence of neurons. β-gal activity is significantly increased in aging hippocampal neurons in vitro [24]. Our results showed that β-gal activity was relatively low in the cortex and hippocampal CA1 and CA3 regions in 6 M and 16 M mice. Compared with younger 6 M mice, β-gal activity was significantly increased in the cortex (Fig. 4A, B; F(3,8) = 52.74, *P* < 0.05 or *P* < 0.01) and hippocampal CA1 (Fig. 4C; F(3,8) = 22.67, *P* < 0.05 or *P* < 0.01) and CA3 (Fig. 4D; F(3,8) = 25.16, *P* < 0.05 or *P* < 0.01) regions in 20 M and 24 M mice, especially in the 24 M group. The results suggested that there was no significant neuronal senescence in 16 M mice, but obvious neuronal senescence was appeared at the age of 20 M and 24 M in mice.

### Effects of aging on MAP2 expression in the cortex and hippocampus in mice

**Fig. 5:**
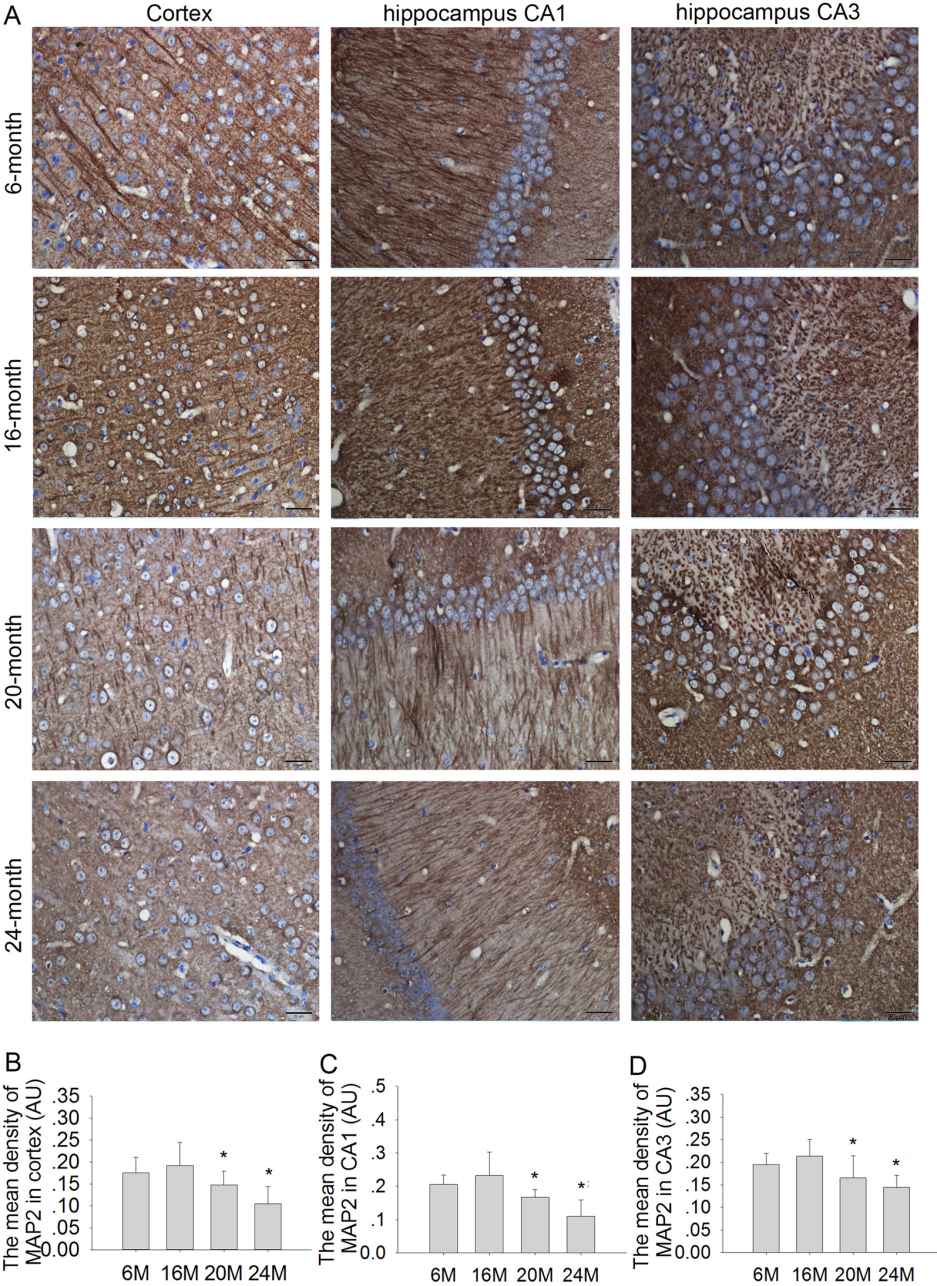
Effects of aging on MAP2 expression in the cortex and hippocampus in mice (immunohistochemistry, 400 × , scale bar = 20 μm). **A** The expression of MAP2 in the cortex, hippocampus CA1 and CA3 in 6 M, 16 M, 20 M and 24 M mice. **B** The mean density of MAP2 in the cortex. **C** The mean density of MAP2 in hippocampus CA1. **D** The mean density of MAP2 in hippocampus CA3. Results are expressed as mean ± SD, n = 4. *P < 0.05, vs the 6 M control

The MAP2 is an important biomarker located in neuronal dendrites. MAP2 expression in the hippocampus and cortex is significantly decreased in old rats [25]. Therefore, we further detected MAP2 expression in the cortex and hippocampal CA1 and CA3 regions using immunohistochemistry. The results showed that the expression of MAP2 was abundant in the cortex and hippocampal CA1 and CA3 regions in 6 M and 16 M mice (Fig. 5). Compared to 6 M mice, the expressions of MAP2 were significantly reduced in the cortex (Fig. 5A, B; F(2,9) = 14.16, *P* < 0.05 or *P* < 0.01) and hippocampal CA1 (Fig. 5C; F(2,9) = 10.67, *P* < 0.05 or *P* < 0.01) and CA3 (Fig. 5D; F(2,9) = 6.263, *P* < 0.05 or *P* < 0.01) regions in 20 M and 24 M mice, especially the 24 M group. These findings suggested that the expression of MAP2 in neurons might significantly decrease when the mice entered older age.

### Effects of aging on NLRP1, ASC, caspase-1, and IL-1β expression in the hippocampus in mice

**Fig. 6:**
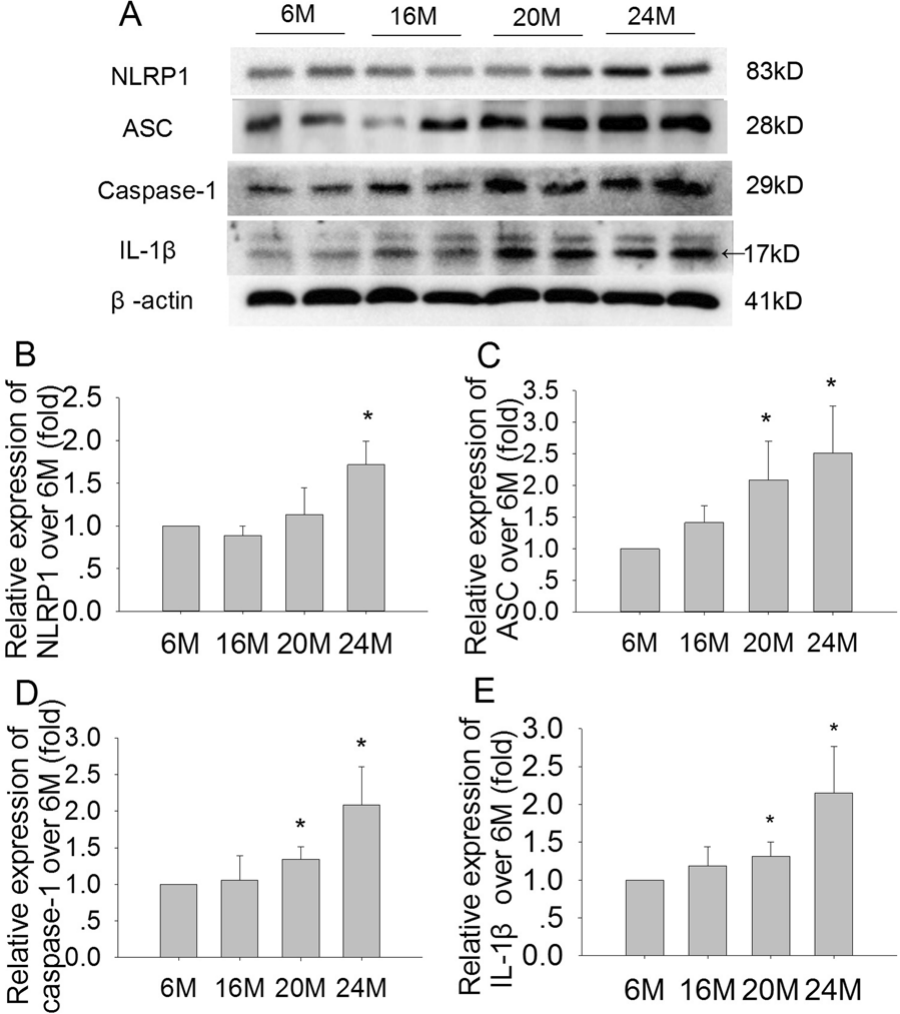
Effects of aging on the expressions of NLRP1, ASC, caspase-1and IL-1β in the hippocampus in mice. **A** The bands of NLRP1, ASC, caspase-1, IL-1β and β-actin examined by immunoblot in 6 M, 16 M, 20 M and 24 M mice. **B** The relative expression of NLRP1 over 6 M. **C** The relative expression of ASC over 6 M. **D** The relative expression of caspase-1 over 6 M. **E** The relative expression of IL-1β over 6 M. Results are expressed as mean ± SD, n = 3. *P < 0.05 vs the 6 M control

In order to confirm whether NLRP1 inflammasome activation is involved in aging-related neuronal damage, we further investigated the expressions of NLRP1, ASC, caspase-1, and IL-1β in the hippocampus. The results showed that the expression of NLRP1 was significantly increased in 20 M and 24 M mice, especially in the 24 M group, compared with 6 M (Fig. 6A, B; F(3,8) = 8.872, *P* < 0.05). In addition, the expressions of ASC, caspase-1, and IL-1β gradually increased with aging; they were significantly increased in 20 M and 24 M mice (ASC: Fig. 6A, C; F(3,8) = 5.362, *P* < 0.05; caspase-1: Fig. 6D; F(3,8) = 7.365, *P* < 0.05; and IL-1β: Fig. 6E; F(3,8) = 6.496, *P* < 0.05) compared to 6 M mice. While in 16 M group, these parameters had no significant changes compared with 6 M mice. The data suggested that NLRP1 inflammasome activation was closely involved in aging-related neuronal damage during aging process.

### Effects of aging on ROS production in the cortex and hippocampus in mice

**Fig. 7:**
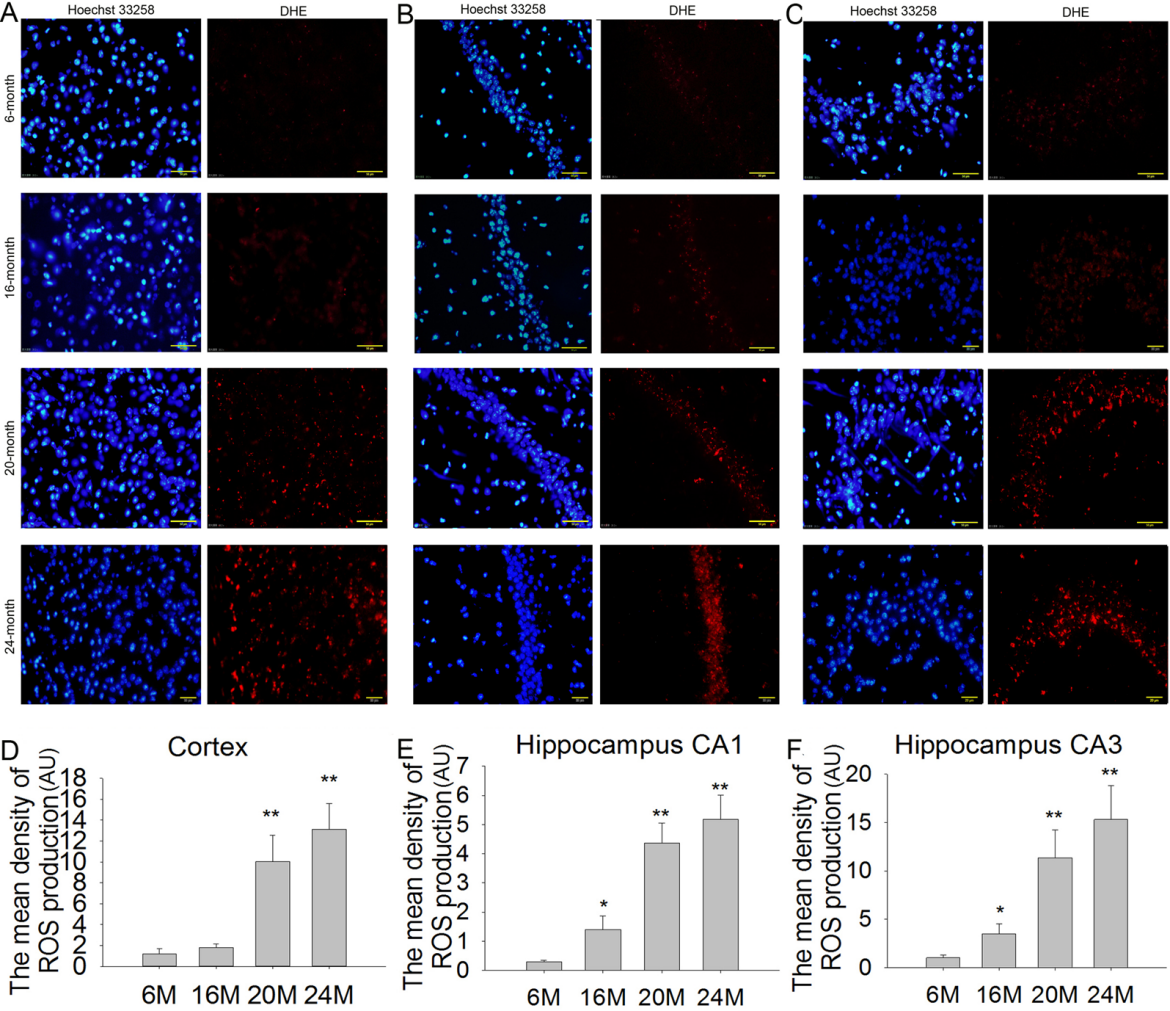
Effects of aging on ROS production in the cortex and hippocampus in mice (DHE staining, 400 × , scale bar = 50 μm). **A**–**C** The ROS production in the cortex, hippocampus CA1 and CA3 in 6 M, 16 M, 20 M and 24 M mice. **D** The mean density of ROS production in the cortex. **E** The mean density of ROS production in hippocampus CA1. **F** The mean density of ROS production in hippocampus CA3. Results are expressed as mean ± SD, n = 3. *P < 0.05, **P < 0.01 vs the 6 M control

Given that ROS plays crucial roles in neuroinflammation and neuronal damage, we also measured ROS production in the cortex and hippocampus via DHE fluorescence staining. The results showed that there was little ROS production in the cortex and hippocampal CA1 and CA3 regions in 6 M mice. In 16 M mice, ROS production was slightly increased in the cortex, but it was significantly increased in hippocampal CA1 (Fig. 7B, E; F(3,8) = 31.36, *P* < 0.05) and CA3 (Fig. 7C, F; F(3,8) = 16.56, *P* < 0.05) regions. In 20 M and 24 M mice, ROS production was significantly increased more than tenfold in the cortex (Fig. 7A, C; F(3,8) = 22.17, *P* < 0.01) and hippocampal CA1 (Fig. 7B, E; F(3,8) = 31.36, *P* < 0.01) and CA3 (Fig. 7C, F; F(3,8) = 16.56, *P* < 0.01) regions compared with 6 M mice. These results suggested that excessive ROS accumulation was closely involved in neuronal damage during aging.

### Effects of aging on NOX2, p22phox, and p47phox expression in the hippocampus in mice

**Fig. 8:**
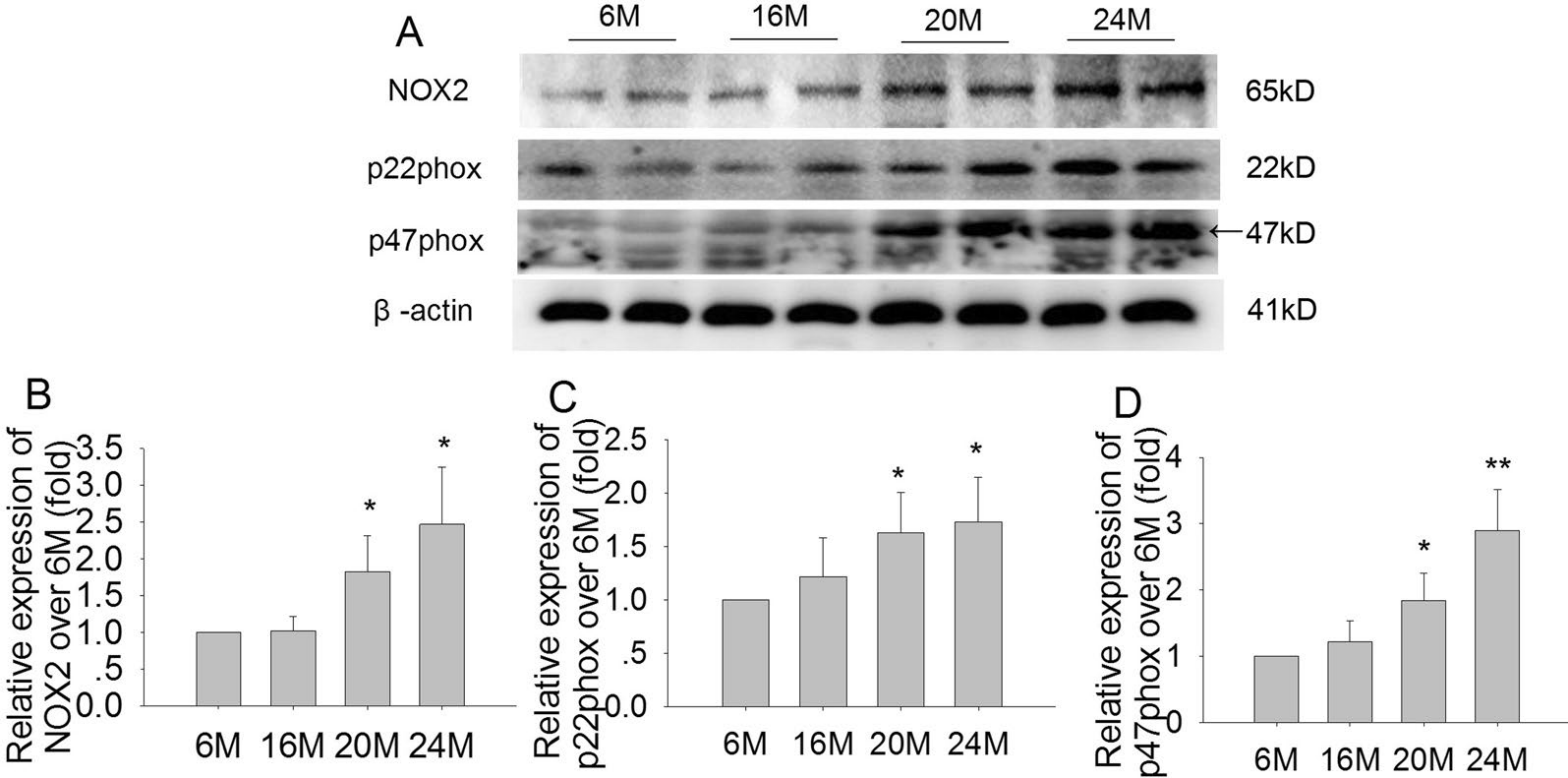
Effects of aging on the expressions of NOX2, p22phox and p47phox in the hippocampus in mice (immunoblot). **A** The bands of NOX2, p22phox, p47phox and β-actin in 6 M, 16 M, 20 M and 24 M mice. **B** The relative expression of NOX2 over 6 M. **C** The relative expression of p22phox over 6 M. **D** The relative expression of p47phox over 6 M. Results are expressed as mean ± SD, n = 3. *P < 0.05, **P < 0.01 vs the 6 M control

The NOX2 is a key enzyme in the process of ROS generation in the brain. Hence, we further measured the effect of senescence on the expressions of NOX2, p22phox, and p47phox proteins in the hippocampus via western blotting. The results showed that the expressions of NOX2, p22phox, and p47phox were relatively low in 6 M and in 16 M mice. Compared with 6 M mice, the expressions of NOX2 (Fig. 8A, B; F(3,8) = 5.791, *P* < 0.05), p22phox (Fig. 8C; F(3,8) = 10.06, *P* < 0.05), and p47phox (Fig. 8D; F(3,8) = 14.75, *P* < 0.05 or *P* < 0.01) were significantly increased in 20 M and 24 M mice. The change of NOX2 was consistent with the ROS production in the brain during aging. These results suggested that NOX2 was closely involved in ROS generation and accumulation in brain during aging.

## Discussion

Aging is an natural, progressive, and deleterious process that can lead to a variety of age-related diseases [26]. Although the basic mechanism of aging is not completely clear, it is easy to show the aging-related changes, such as the decline in the function of various organs and systems. The brain is not an exception. Significant cognitive decline and neuronal damage are observed with brain senescence [27]. As the mechanism of aging has not been fully elucidated and there are still no effective anti-aging measures, it is very important to study the mechanisms that underlie neuronal damage during aging. Furthermore, it remains unclear when the brain begins to show obvious age-related damages during the course of aging. In the current study, we observed the correlation between aging-related neuronal damage and alterations in the NLRP1 inflammasome in the cortex and hippocampus in young (6 M) and older (16 M, 20 M, and 24 M) mice. The current study demonstrated a significant increase in β-gal activity and neuronal damage in aged mice, particularly 20 M and 24 M mice. These changes were accompanied by a significant reduction in locomotor activity and learning and memory functions. Consistent with the pathological changes in the brain, the expressions of NOX2 and NLRP1 inflammasome were significantly increased in the hippocampus in the older mice, especially 20 M and 24 M mice. Our results suggested that the NLRP1 inflammasome might play an important role in aging-related neuronal damage.

Aging is associated with cognitive impairment, and brain regions (including the cortex and hippocampus) that are responsible for learning and memory are particularly vulnerable to aging [28]. The OFT can measure exploration, anxiety, and locomotor behavior of animals [29]. The MWM is an important method to detect learning and memory impairment, which is sensitive to hippocampal damage [30]. Previous studies have revealed the changes of learning and memory function in young and old animals, but there is little research on how the learning and memory function changes with age. Gil-Mohapel et al. [31] reported that hippocampal neurogenesis affects the type of search strategies with age, while the ability to learn the task is not further influenced by the age-induced decrease in neurogenesis after 1.5 months of age in mice. In the study, we found that there was no significant difference in the learning test in 16 M compared to 6 M mice. However, in 20 M and 24 M mice, especially the 24 M group, severe declines in motor and exploratory abilities and learning and memory functions were observed. Gil-Mohapel et al. proposed that pre-existing neurons may compensate for the reduction in neurogenesis, but they only detected the learning ability in 1.5–12-month-old mice [31]. We found that there were significant learning and memory impairments in 20 M and 24 M mice. These changes may be induced by neuronal damages in the cortex and hippocampus. Hence, to confirm whether the behavioral changes are consistent with the neuronal pathological changes in aging process, we further observed the pathological changes in the cortex and hippocampal CA1 and CA3 regions in 6 M, 16 M, 20 M, and 24 M mice. H&E and Nissl staining results suggested that 16 M mice had no obvious neuronal damages, while in 20 M and 24 M mice, there were significant neuronal damages in the cortex and hippocampus especially in the 24 M mice, the pyknotic cells were significantly increased.

β-gal is an important biomarker for cell aging, during aging. We found that the expression of β-gal had no significant increase in 6 M and 16 M mice. However, the β-gal expression was dramatically increased in 20 M and 24 M mice in the cortex and hippocampal CA1 and CA3 regions, especially in 24 M mice. These results echo previous research [32] and suggest that the age of 16–20 months may be an important period during which aging-related neuronal damage occurs in mice. MAP2 is a cytoskeletal and neuronal marker protein. The expression of MAP2 is consistent with dendritic growth, branching, and late dendritic remodeling [33]. The expression of MAP2 is significantly reduced in the hippocampus and cortex in aged rats [25]. However, to date no study has systematically investigated how MAP2 expression varies with age. In this study, we found that the expression of MAP2 had no significant changes in the presenile 16 M mice compared with the younger 6 M mice. While in aged 20 M and 24 M mice, MAP2 was significantly decreased, and this phenomenon is consistent with neuronal damage in aging mice. Therefore, we speculated that the expression of MAP2 is not significantly decreased until the neurons show obvious aging-related damage.

In recent years, increasing evidence has suggested that neuroinflammation is an important event in brain aging and senescence-associated neuronal damage [12]. Neuronal inflammation also contributes to the pathogenesis of brain aging and neurodegenerative diseases [34]. It has been reported that proinflammatory cytokines released from microglia and astrocytes significantly induce neuronal damages and apoptosis in AD [35]. Recent studies have shown that, in addition to microglia and astrocytes, neurons also contribute to the inflammatory response in the brain by releasing cytokines, such as IL-1β and IL-18 [36]. Inflammasome is a multi-protein complex in the cytoplasm that is involved in maturation of proinflammatory molecules such as IL-1β, IL-6, and IL-18. NLRP1 is the first reported member of the NLRP family to form an inflammasome, and is expressed ubiquitously in the brain, particularly in neurons [6, 37]. The NLRP1 inflammasome play an important role in many neurological diseases, and studies in AD models indicate that the NLRP1 inflammasome is significantly upregulated in neurons [37]. The NLRP1 inflammasome is composed of NLRP-1, procaspase-1, and the adaptor protein ASC [38]. Caspase-1 activation is initiated by NLRP1, and ASC can enhance caspase-1 activity, which mediates the maturation of IL-1β and IL-18 to trigger inflammation [39]. We speculate that the NLRP1 inflammasome is involved in aging-related neuronal damage in aging process. The present study demonstrated that there was no significant difference in the expressions of NLRP1, ASC, caspase-1, and IL-1β in 6 M and 16 M mice. While in 20 M and 24 M mice, the expressions of NLRP1, ASC, caspase-1 and IL-1β were significantly increased, especially in 24 M mice. These changes of NLRP1 inflammasome were consistent with the results of neuronal damage during the aging process in mice.

It is well known that excessive ROS production is one of the main causes of NLRP1 inflammasome activation. Increasing evidence has suggested that excessive ROS-induced oxidative stress and neuroinflammation are important events in brain aging and senescence-associated neuronal damage [12]. ROS-induced oxidative stress has been recognized as a contributing factor in aging and the progression of multiple neurodegenerative diseases such as AD [40]. ROS production is increased in aging mouse brain in association with significant cognitive impairment, and SOD mimetic treatment reduces age-associated oxidative stress, significantly extended lifespan, and improves learning and memory performance [41]. Therefore, we speculated that excessive ROS production might involve in NLRP1 inflammasome activation in neurons during aging process. In the present study, we found that only a small amount of ROS was produced in the cortex and hippocampus of 6 M mice. The ROS production was slightly increased in 16 M mice, and the relative expressions of NOX2, p22phox, and p47phox were also slightly increased in CA1 and CA3, but the difference has no significance. We thought that there were other factors involved in ROS accumulation such ROS scavenging system, which requires further study. But in 20 M and 24 M mice, the ROS production in the cortex and hippocampus was significantly increased over 10 times than that of in 6 M mice. These data suggest that excessive ROS accumulation is closely involved in the brain aging process. Brain ROS production was significantly increase from 16 to 20 M; hence, this period might be important for preventing brain aging.

Many enzymes are involved in intracellular ROS generation. Among them, the NADPH oxidase (NOX) is currently the only enzyme family known to produce ROS as its sole function. NOX is composed by several isoforms such as NOX1-5. NOX2 is constitutively expressed in many cells in the brain, especially neurons [42], and contributes to superoxide production in the cerebral circulation under physiological conditions [43]. It has been well elucidated that NOX2 activation plays an important role in the pathogenesis of neurodegenerative diseases, such as AD and PD [44]. However, the change of NOX2 in brain aging process remains unclear. The present results showed that, consistent with the ROS production, the expressions of NOX2, p22phox, and p47phox only exhibited an increased trend in 16 M mice. By contrast, in 20 M and 24 M mice, the expressions of NOX2, p22phox, and p47phox were significantly increased compared to 6 M mice. These findings confirm that NOX2 plays an important role in ROS generation during brain aging, and redox imbalance is closely involved in aging-related neuronal damage during aging process. Additionally, the NOX2 expression and ROS production were consistent with the results of aging-related neuronal damage and NLRP1 inflammasome activation during the brain aging process.

Above all, brain aging is a complex process that changes with time. During aging, the function of neurobiological networks also showed obvious damage in elderly mice, especially in 24 M mice. A large number of studies have shown that there are significant changes in molecules, cells, brain regions and learning and memory during aging. In the present study, we found that ROS production and NOX2 enzyme systems, that promotes ROS production, were significantly increased; Inflammatory cytokines and NLRP1 inflammasome were also significantly increased in elderly mice, especially in 24 M mice. The excessive increase of these substances further damages the structure and function of neurons. The cortex and hippocampus are important neurobiological networks involved in learning and memory. We found that the neurons showed obvious degeneration, and the Nissl bodies and MAP2 expression were significantly decreased in the cortex and hippocampus CA1 and CA3 regions. These changes of neurobiological networks eventually lead to learning and memory impairments in elderly mice, especially in 24 M mice.

In conclusion, our study suggested that NOX2-NLRP1 inflammasome signaling is closely involved in aging-related neuronal damage during the aging process, and is perhaps an important target for modulating brain aging. However, there were many limitations of the methods in our study. We only observed the changes of 6 M, 16 M, 20 M and 24 M mice in the process of aging, perhaps more time points are needed in the future study. And this study did not provide direct evidence for the role of NOX2-NLRP1 signaling in brain aging, the precise mechanisms of NLRP1 inflammasome during brain aging warrant further investigation.

## Supplementary Information

**Additional file 1: Fig S1.** The experimental procedure of this study. The full-term mice (6 M, 16 M, 20 M, 24 M) were allowed 24 h to adjust the environment, and performed the OFT on the second day (day2), followed by orientation navigation experiment (day3-day6) and space exploration experiment (day7) of the MWM. Then, the mice were sacrificed (day8) and the brain tissues were processed for other tests.

**Additional file 2: Fig S2.** Effects of aging on pathological changes in the cortex and hippocampus in mice (n = 4, H&E staining, 400 × , scale bar = 20 μm). Black arrows indicate nuclear pyknosis and hyperchromatic nuclei. Yellow arrows indicate eosinophilic degeneration.

## Data Availability

The datasets used and analysed during the present study are available from the corresponding author on reasonable request.
