# Supplementary figures and images for: NLRP1 inflammasome involves in learning and memory impairments and neuronal damages during aging process in mice
Source: Behav Brain Funct. 2021 Dec 17;17:11. doi: 10.1186/s12993-021-00185-x (PMC8680336; doi:10.1186/s12993-021-00185-x)

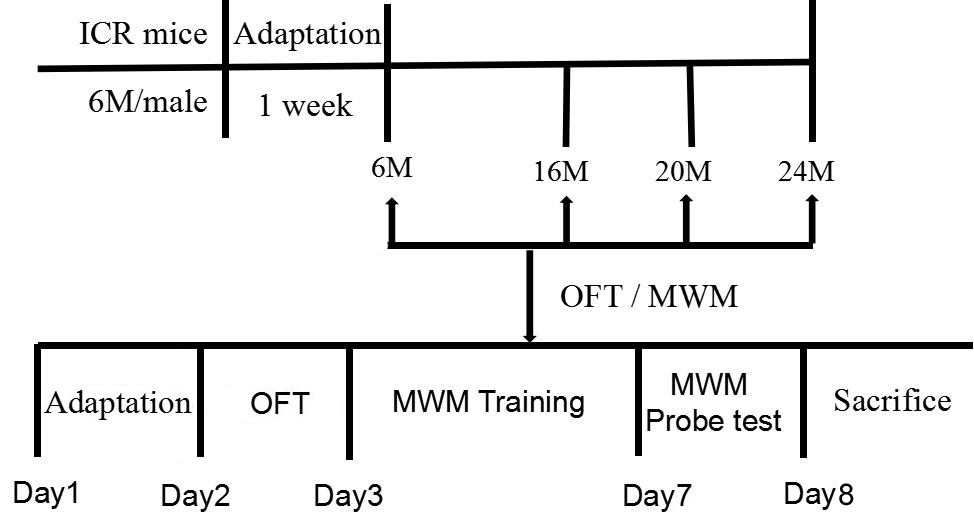

Supplement: Supplementary file 1 — Additional file 1: Fig S1. The experimental procedure of this study. The full-term mice (6 M, 16 M, 20 M, 24 M) were allowed 24 h to adjust the environment, and performed the OFT on the second day (day2), followed by orientation navigation experiment (day3-day6) and space exploration experiment (day7) of the MWM. Then, the mice were sacrificed (day8) and the brain tissues were processed for other tests. [file 12993_2021_185_MOESM1_ESM.jpg]

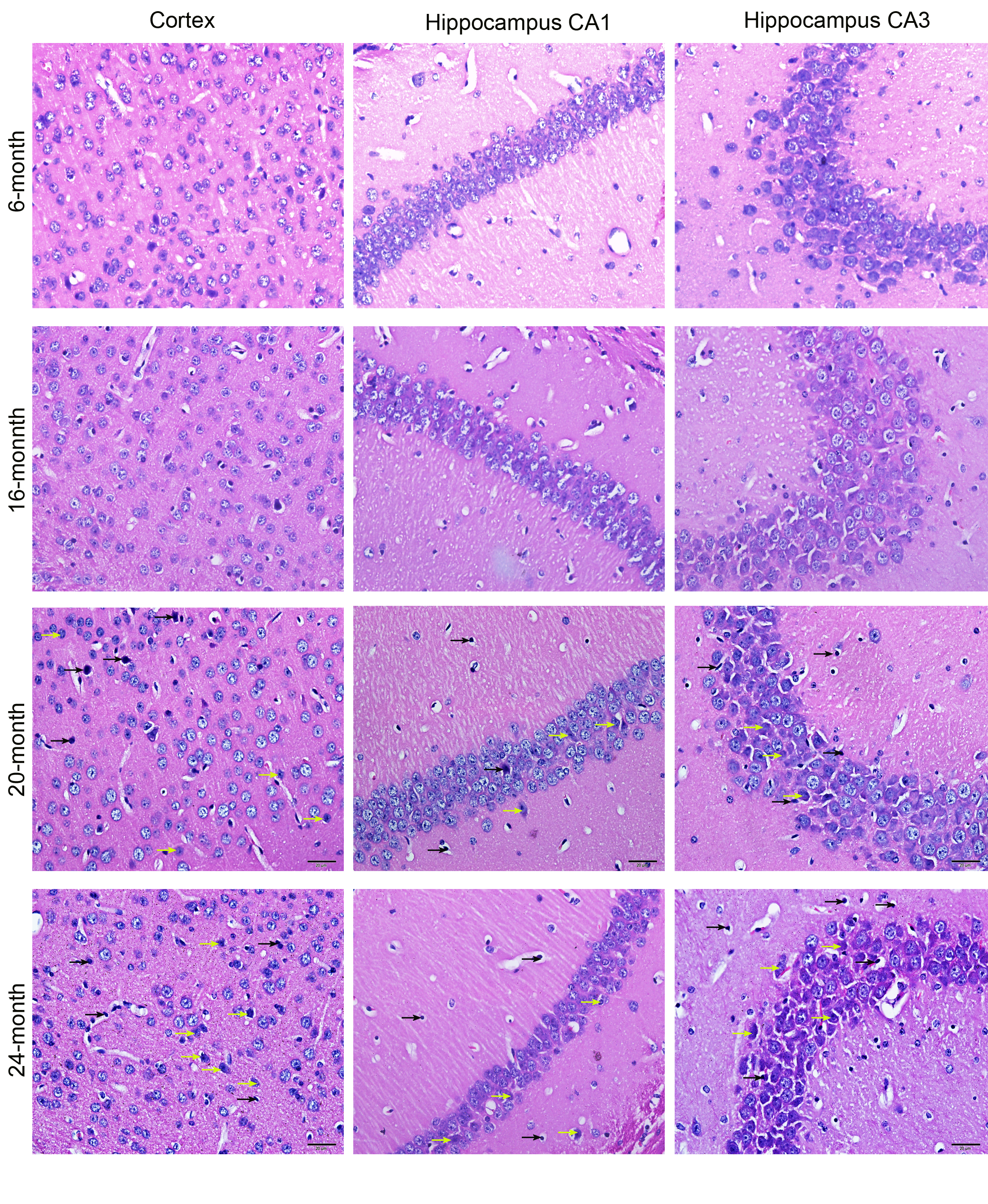

Supplement: Supplementary file 2 — Additional file 2: Fig S2. Effects of aging on pathological changes in the cortex and hippocampus in mice (n = 4, H&E staining, 400 × , scale bar = 20 μm). Black arrows indicate nuclear pyknosis and hyperchromatic nuclei. Yellow arrows indicate eosinophilic degeneration. [file 12993_2021_185_MOESM2_ESM.jpg]
